# Association between Olaparib Exposure and Early Toxicity in BRCA-Mutated Ovarian Cancer Patients: Results from a Retrospective Multicenter Study

**DOI:** 10.3390/ph14080804

**Published:** 2021-08-16

**Authors:** Maud Velev, Alicja Puszkiel, Benoit Blanchet, Sixtine de Percin, Nicolas Delanoy, Jacques Medioni, Claire Gervais, David Balakirouchenane, Nihel Khoudour, Patricia Pautier, Alexandra Leary, Zahra Ajgal, Laure Hirsch, François Goldwasser, Jerome Alexandre, Guillaume Beinse

**Affiliations:** 1 Department of Medical Oncology, Cochin University Hospital, Assistance Publique-Hôpitaux de Paris, 75014 Paris, France; Maud.velev@etu.upmc.fr (M.V.); sixtine.depercin@aphp.fr (S.d.P.); zahra.ajgal@hotmail.fr (Z.A.); laure.hirsch@aphp.fr (L.H.); François.goldwasser@aphp.fr (F.G.); guillaume.beinse@aphp.fr (G.B.); 2 Department of Pharmacokinetics and Pharmacochemistry, Cochin University Hospital, Assistance Publique-Hôpitaux de Paris, 75014 Paris, France; alicja.puszkiel@aphp.fr (A.P.); benoit.blanchet@aphp.fr (B.B.); David.Balakirouchenane@aphp.fr (D.B.); Nihel.Khoudour@aphp.fr (N.K.); 3 INSERM UMR-S1144, Faculté de Pharmacie, Université de Paris, 75006 Paris, France; 4 UMR8038 CNRS, U1268 INSERM, Faculté de Pharmacie, Université de Paris, PRES Sorbonne Paris Cité, CARPEM, 75006 Paris, France; 5 Department of Medical Oncology, Georges Pompidou European Hospital, Assistance Publique-Hôpitaux de Paris, 75015 Paris, France; Nicolas.Delanoy@aphp.fr (N.D.); Jacques.Medioni@aphp.fr (J.M.); Claire.Gervais@aphp.fr (C.G.); 6 Gustave Roussy Cancer Center, Department of Medical Oncology, Université Paris-Saclay, 94805 Villejuif, France; Patricia.PAUTIER@gustaveroussy.fr (P.P.); Alexandra.LEARY@gustaveroussy.fr (A.L.); 7 Centre de Recherche des Cordeliers, Université de Paris, Sorbonne Université, Inserm, Team Personalized Medicine, Pharmacogenomics and Therapeutic Optimization (MEPPOT), 75006 Paris, France

**Keywords:** olaparib, population pharmacokinetics, PK-toxicity relationship, ovarian cancer, therapeutic drug monitoring

## Abstract

Factors associated with olaparib toxicity remain unknown in ovarian cancer patients. The large inter-individual variability in olaparib pharmacokinetics could contribute to the onset of early significant adverse events (SAE). We aimed to retrospectively analyze the pharmacokinetic/pharmacodynamic relationship for toxicity in ovarian cancer patients from “real life” data. The clinical endpoint was the onset of SAE (grade III/IV toxicity or dose reduction/discontinuation). Plasma olaparib concentration was assayed using liquid chromatography at any time over the dosing interval. Trough concentrations (CminPred) were estimated using a population pharmacokinetic model. The association between toxicity and clinical characteristics or CminPred was assessed by logistic regression and non-parametric statistical tests. Twenty-seven patients were included, among whom 13 (48%) experienced SAE during the first six months of treatment. Olaparib CminPred was the only covariate significantly associated with increased risk of SAE onset (odds ratio = 1.31, 95% CI = [1.10; 1.57], for each additional 1000 ng/mL). The ROC curve identified a threshold of CminPred = 2500 ng/mL for prediction of SAE onset (sensitivity/specificity 0.62 and 1.00, respectively). This study highlights a significant association between olaparib plasma exposure and SAE onset and identified the threshold of 2500 ng/mL trough concentration as potentially useful to guide dose adjustment in ovarian cancer patients.

## 1. Introduction

Olaparib is an oral poly(ADP-ribose) polymerase (PARP) inhibitor [1]. By inhibiting PARP1 and PARP2, enzymes involved in DNA single-strand breaks (SSB) repair, olaparib leads to accumulation of SSB and subsequent deleterious double-strand breaks (DSB). While a cell with an intact homologous recombination (HR) pathway can repair these DSB effectively, olaparib causes synthetic lethality in HR deficient tumor cells, such as in *BRCA1/2*-mutated cancers [2]. Olaparib was therefore initially developed for the treatment of HR-deficient cancers in patients carrying *BRCA1/2* mutation [3]. Beyond *BRCA1/2* mutations, a number of studies showed a survival benefit in patients affected by HR-deficient ovarian, breast, prostate or pancreatic cancers [4,5,6,7,8,9,10].

Clinical studies reported incidences of serious adverse events and dose reduction/discontinuation of 21% and 30%, respectively, in maintenance therapy for newly diagnosed patients [8], and of 35% and 28%, respectively, in platinum-sensitive ovarian cancer relapse [10]. The most common toxicities were digestive (nausea, vomiting), hematological (anemia) and asthenia. In a meta-analysis of olaparib safety from four randomized clinical trials data, Ricci et al. reported an overall incidence of grade III-IV adverse events of 41% [11]. However, despite this high frequency of significant adverse events, factors associated with olaparib toxicity remain largely unknown, supporting the need for reports from “real-life” patients [12,13].

Because of a wide inter-patient pharmacokinetic (PK) variability observed in the last decade with oral antineoplastic agents, therapeutic drug monitoring (TDM) emerged as an important tool [14,15]. Many factors have been shown to significantly influence olaparib exposure. Food has been shown to delay olaparib absorption resulting in a significant decrease in peak plasma concentrations (C_max_) but the impact on area under the concentration–time curve (AUC) is only marginal [16]. In vitro studies suggested that large variations of serum albumin concentrations may impact the unbound fraction of olaparib and subsequently increase the toxicity. Olaparib is mainly metabolized via CYP3A4/5 isoenzymes and co-administration of a potent CYP3A4/5 inhibitor or inducer could also influence olaparib exposure [17]. Finally, impaired renal function has been associated with increased exposure to olaparib [18]. Of note, a new potential source of variability emerged with the approval of a new formulation (capsule and tablets, capsule being now withdrawn from the market) [19].

The PK of olaparib has been assessed by nonlinear mixed-effects modeling using clinical trials’ data [19]. However, the inter-individual variability in olaparib plasma exposure in non-selected real-life ovarian cancer patients has never been assessed [17,19] and little is known about its pharmacokinetic/pharmacodynamic (PK/PD) relationship for toxicity.

In the present retrospective multicenter study, we aimed to investigate the association between olaparib toxicity and potential explanatory factors, such as patient characteristics and olaparib plasma exposure, in patients treated for ovarian cancer in a real-life setting.

## 2. Results

### 2.1. Patient Characteristics

**Figure 1:**
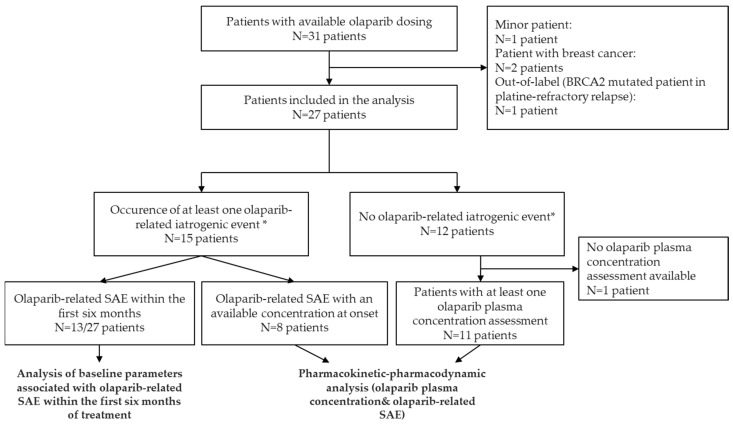
Flow chart. * Olaparib-related iatrogenic adverse events were considered as any clinically significant adverse event (SAE) defined by: (i) grade III–IV adverse events, or (ii) adverse events resulting in dose reduction or treatment discontinuation.

Among 31 patients for whom at least one olaparib plasma concentration was available between November 2016 and August 2020, 4 were excluded (age < 18 years old: N = 1; breast cancer: N = 2; out-of-label use: N = 1). Overall, 27 patients were included in the statistical analysis (Figure 1). These patients were followed in three University Hospitals: Cochin Hospital (Paris, N = 16), Georges Pompidou European Hospital (Paris, N = 6), and Gustave Roussy Cancer Campus (Villejuif, N = 5). Table 1 presents patient and cancer characteristics. The median age at diagnosis was 63 years. Six patients (22%) had an ECOG-PS = 2 at olaparib initiation. None had residual biological toxicities beyond grade I from previous treatments. Ten patients (62%) had renal insufficiency (Cockcroft clearance < 60 mL/min) at baseline, including 2 patients with Cockcroft–Gault clearance of 30–50 mL/min who were treated at full dose. Except for one 85-year-old patient who received an initial dose of 200 mg tablet bid, all patients received the initial recommended dose of 400 mg bid for capsules (N = 16) or 300 mg bid for tablets (N = 10).

### 2.2. Pharmacokinetic Analysis

Overall, 66 plasma concentrations were analyzed in 27 patients. Among these, three samples were excluded (N = 3 patients) because of unavailable data on delay between olaparib intake and sampling, and two (N = 2 patients) because of a delay of more than 24 h. Finally, 61 plasma concentrations were included in the PK analysis (N = 22 patients). The median number of concentrations per patient was 2 (range: 1–7) with a median sampling time of 10 h post-dose (range: 0.75–24).

**Figure 2:**
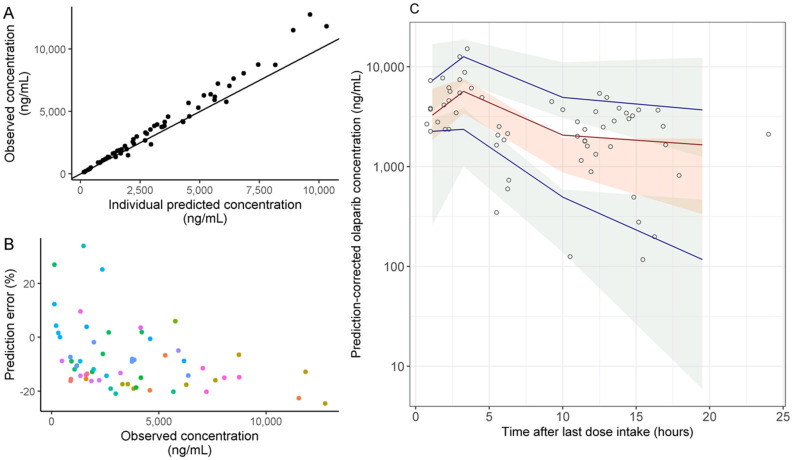
Evaluation of the predictive performance of the population PK model. (**A**) Observed vs. individual predicted olaparib concentrations. Solid line represents the identity line (y = x) (**B**) Prediction error (PE) vs. observed concentrations. (**C**) Prediction-corrected visual predictive check (pcVPC) for olaparib. pcVPC obtained by N = 1000 simulations of the original dataset with the mean parameters (fixed and random effects). The shaded areas represent the 95% confidence intervals around the 5th, 50th (median) and 95th percentile of the simulated concentrations, the lines represent the 5th, 50th (median) and 95th percentile of the observed concentrations and the circles represent the observed concentrations.

A two-compartment population PK model was applied for the analysis of the concentration–time data. The median prediction error (PE) was −11.9% [interquartile range, IQR = −15.9; −4.9] showing good accuracy and precision of the model predictions. Figure 2A,B present observed vs. predicted concentrations and PE vs. observed concentrations. The prediction-corrected visual predictive check (pcVPC) plot showed good agreement between simulated and observed olaparib plasma concentrations (Figure 2C). Although a slight underestimation of the observed concentrations was observed, the number of concentrations with absolute PE < 20% and <30% was 53 (86.9%) and 60 (98.4%), respectively, showing that the observed concentrations were satisfactorily well predicted.

**Figure 3:**
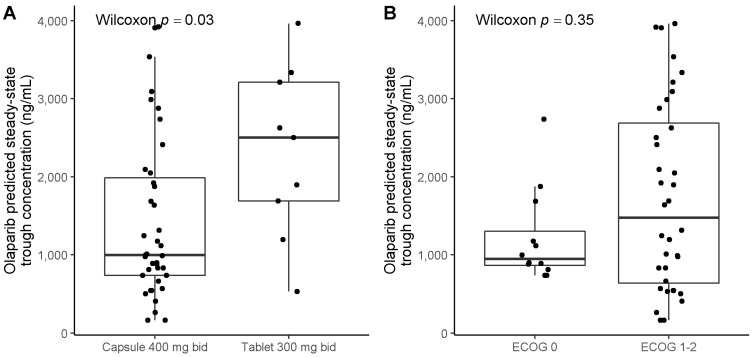
Olaparib steady-state predicted trough concentrations (C_minPred_) according to olaparib formulation and ECOG-PS. (**A**) C_minPred_ according to olaparib dose and formulation. (**B**) C_minPred_ according to ECOG-PS. ECOG-PS: Eastern Cooperative Oncology Group Performance Status. Patients included in this analysis are patients treated with the recommended dose.

The median [IQR] predicted trough concentration (C_minPred_) was 1241 ng/mL [881–2412 ng/mL] in the entire study population. In patients treated with the recommended dose, the median [IQR] C_minPred_ was 997 ng/mL [737–1985 ng/mL] and 2503 ng/mL [1688–3213 ng/mL], for those receiving 400 mg bid capsule (N = 39 concentrations) and 300 mg bid tablets (N = 9 concentrations), respectively (Figure 3A). C_minPred_ were not significantly different according to ECOG-PS (Figure 3B). The inter-individual variability in C_minPred_ (coefficient of variation, CV%) was 64% and 53% for capsule and tablet formulation, respectively (when considering 1st plasma concentration for patients treated with the recommended dose, N = 22).

**Figure A1:**
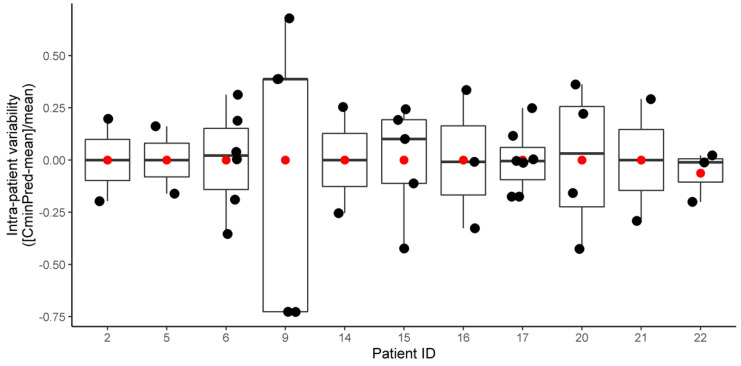
Intra-individual variability in olaparib C_minPred_ calculated as % deviation of each individual C_minPred_ from the mean concentration over the treatment period for the same individual (N = 11 patients with more than one olaparib exposure assessment and treated at standard dose).

Intra-patient variability was evaluated by the deviation from each C_minpred_ to mean C_minPred_ within each patient (N = 11 patients with more than one olaparib exposure assessment and treated at standard dose). Median [IQR] absolute intra-individual variability was 19.7% [11.6–32.7%]. (Figure A1).

### 2.3. Association between Patients’ Baseline Characteristics and Onset of Clinically Significant Adverse Events (SAE)

During the first six months of treatment, 13 patients (48%) experienced a SAE: (i) seven patients experienced a grade III–IV adverse event (anemia N = 5; asthenia N = 1; skin rash N = 1); (ii) six patients experienced an adverse event resulting in dose reduction or discontinuation (digestive: N = 3; asthenia: N = 2; anemia: N = 1). The median time to SAE onset was 2 months [0.9–2.1]. Patients’ baseline characteristics and galenic formulation were not associated with the onset of SAE in the univariate analysis (Table 2).

### 2.4. Association between Olaparib Exposure and Toxicity

**Figure 4:**
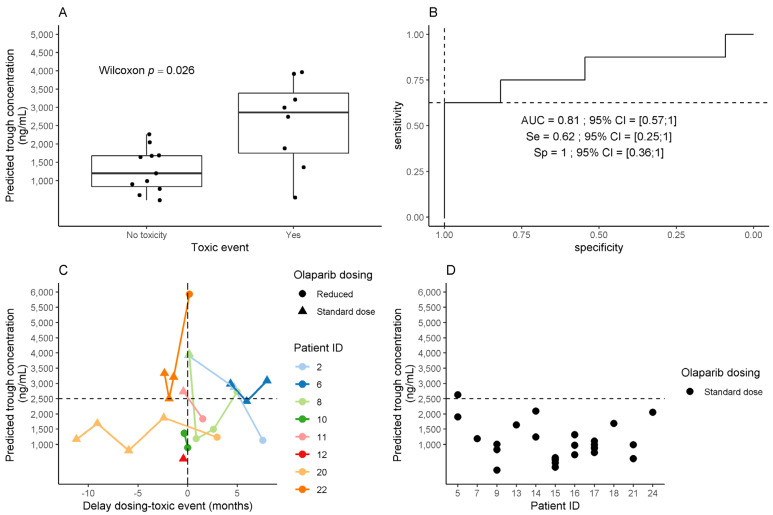
Association between olaparib predicted trough concentration (C_minPred_) and onset of olaparib-related clinically significant adverse events (SAE). The black dashed line indicates the threshold identified by ROC curve analysis (2500 ng/mL). (**A**) Olaparib C_minPred_ in patients with or without olaparib-related SAE. Toxic event refers to SAE onset. (**B**) Performances of olaparib C_minPred_ for prediction of olaparib-related SAE (ROC curve). Se: sensitivity. Sp: specificity. AUC: area under curve. The 95% confidence intervals were computed by 2000 stratified bootstrap replicates. (**C**) Olaparib C_minPred_ in patients who experienced a SAE. Patients included in this analysis are patients who experienced a SAE, with an olaparib exposure assessment at SAE onset (+/− 6 months around SAE). (**D**) Olaparib predicted trough concentration (C_minPred_) in patients without olaparib-related adverse event. Patients included in this analysis are patients with at least one plasma concentration available, and no adverse event within the six months around.

Among 22 patients from the PK analysis, 19 were included in the PK-toxicity analysis. Three patients were excluded because the delay between SAE and measurement of olaparib plasma concentration was more than 6 months. Among these 19 patients, 8 experienced SAE over the treatment course (including two patients with SAE beyond 6 months after olaparib initiation: anemia at 12 months and digestive toxicity at 26 months) and 11 patients did not experience SAE. The delay between the SAE occurrence and the measurement of olaparib plasma concentrations was 2 to 4 months (Figure 4C).

Patients who experienced SAE had higher median plasma olaparib C_minPred_ than other patients (2862 ng/mL vs. 1195 ng/mL, respectively; *p* = 0.026) (Figure 4A). Increased C_minPred_ was associated with a higher risk of SAE, with an odds ratio of 1.31 (95% CI = 1.10–1.57) for each additional 1000 ng/mL. C_minPred_ was found predictive of SAE onset in the ROC analysis with an AUC of 0.81 (95% CI = 0.57–1.00) (Figure 4B). Based on the distribution of C_minPred_ in the studied population and sensitivity/specificity in the ROC analysis, a threshold of 2500 ng/mL was selected as the lowest (i.e., most sensitive) threshold at specificity = 100% (Figure 4B). At this threshold, only one of 11 patients without SAE had a C_minPred_ above 2500 ng/mL, vs. 5 of 8 patients who experienced an olaparib-related SAE (Figure 4C,D) (9.1 vs. 62.5%, respectively; Chi2 test, *p* = 0.01).

Among the three patients who experienced an SAE and exhibited C_minPred_ below 2500 ng/mL, two of them had a dose reduction in the first month of olaparib treatment for grade II asthenia while being ECOG-PS = 2 at baseline (patient #10: 73 years old patient with albuminemia 46 g/L; patient #12: 85 years old patient with 39 g/L albuminemia). Patient #20 experienced a grade II anemia resulting in dose reduction, controlled thereafter with a C_minPred_ at 1241 ng/mL with a grade I anemia.

### 2.5. Pharmacokinetic Drug–Drug Interactions

Among 27 patients, 4 (15%) had a potential drug interaction (PDI) which could result in olaparib over-exposure. Three patients were concomitantly treated with CYP3A4/5 inhibitors at baseline: amiodarone (N = 2), aprepitant (N = 1). These three patients experienced toxicity within three months. According to DDI predictor, the mean predicted increase in plasma olaparib exposure (AUC) is 1.39 (95% CI = 0.99–1.97) and 2.07 (95% CI = 1.30–3.29]) for amiodarone 1200 mg/day and aprepitant 80 mg/day, respectively.

Patient #8 (Figure 4C) treated with amiodarone 200 mg daily for severe amyloidosis cardiomyopathy had several available PK samples: (i) C_minPred_ of 3964 ng/mL at SAE onset, 1 month after olaparib treatment start (tablet 300 mg bid), was associated with an episode of asthenia grade II, leading to a dose reduction to 150 mg bid; (ii) one month after olaparib dose reduction, C_minPred_ was 1649 ng/mL, and (iii) four months later, at another acute event occurrence (acute pyelonephritis with bacteriemia), olaparib C_minPred_ was 2732 ng/mL. Olaparib was held during the acute event, and reintroduced after resolution at the same dose. No further olaparib plasma concentration assessment was performed for this patient.

Patient #2 (Figure 4C) was treated for atrial fibrillation cardioversion with a high intravenous dose of amiodarone after 26 months of olaparib treatment (capsule 400 mg bid). She experienced a grade II digestive toxicity two months after amiodarone introduction. Olaparib C_minPred_ at SAE onset was 3919 ng/mL. After 5 months of amiodarone discontinuation, olaparib C_minPred_ was still increased (2876 ng/mL) without any modification of olaparib dose. The long terminal half-life of amiodarone (9–77 days [20]) could explain this slow decrease in olaparib C_minPred_. In this context, olaparib dose was finally reduced to 100 mg bid. C_minPred_ was 1133 ng/mL after 20 days of dose reduction.

## 3. Discussion

Our study is the first to comprehensively analyze factors potentially leading to olaparib SAE. We observed a significant relationship between plasma olaparib exposure and SAE onset, leading us to propose a threshold of plasma trough concentration > 2500 ng/mL as significantly associated with an increased risk of SAE onset.

In our study, blood samples for plasma drug monitoring were collected at any time after dose intake. Thereby, we used a previously published population PK model for olaparib to estimate trough concentrations [17] to avoid bias in the PK-toxicity analysis related to the variability in the sampling time. The predictive performance of the model when applied to real-life data was evaluated by calculation of PE and simulation-based pcVPC. The median PE was −11.9% [IQR = −15.9; −4.9] showing that the observed and predicted concentrations were in good agreement although a slight underestimation of the concentrations was observed. The pcVPC showed good predictive performance of the model when applied to our data. However, the median of the observed concentrations was slightly higher than the prediction interval at 10 h post-dose. This might be due to the low number of patients and sparse PK data in our study and potential enrichment in concentrations in patients presenting a toxic event. Thus, olaparib plasma concentrations in our cohort might be higher than those observed in clinical trials used to develop the PK model by Zhou et al. Nevertheless, when considering absolute PE values, 86.9% and 98.4% of concentrations had an absolute PE <20% and <30%, respectively. In addition, the median PE was lower than the median intra-individual variability in C_minPred_. Taken together with all these arguments, C_minPred_ can be considered as a reasonable estimation of the trough exposure in our study. Furthermore, the median C_minPred_ for 400 mg bid capsules and 300 mg bid tablets (997 ng/mL and 2503 ng/mL, respectively) were in accordance with values previously reported from clinical trials data (mean steady-state C_min_ of 1290 ng/mL (CV = 133%) and 1840 ng/mL (CV = 67%) for capsule 400 mg bid and tablet 300 mg bid, respectively) [21]. However, a slightly higher C_minPred_ was observed in patients treated with 300 mg tablets compared to the literature data. This could be explained by enrichment in olaparib concentration assessment at SAE onset. Overall, our external validation of the PK model allowed using it for the prediction of trough concentrations, which also suggests that this model could be used in daily clinical practice. Despite the selection bias due to the exclusive consideration of patients for whom an olaparib exposure assessment was performed, the rate of clinically significant adverse events in our population (48%) was consistent with the rate of 41% of grade III-IV adverse events previously reported [11].

To date, factors associated with a higher risk of SAE have not been clearly identified in ovarian cancer patients from the “real world”. Additionally, the olaparib PK/PD relationship in this population has not yet been evaluated. In the present study, increased olaparib C_minPred_ was the only factor significantly associated with a higher risk of SAE occurrence (OR = 1.31, 95% CI = 1.10–1.57). As far as we know, no such relation was previously reported in patients from clinical trials. In this study, we focused on a non-selected real-life population which is likely more fragile than that included in clinical trials. This results in both greater sensitivity to the induced-olaparib toxic effects and in a larger inter-individual variability in the olaparib PK. These two elements could contribute in part to explaining the discrepancy of our results with those reported in the literature. In order to help physicians in their therapeutic decision, we decided to determine C_minPred_ threshold predicting the occurrence of SAE with a specificity of 100%. In this context, a threshold of 2500 ng/mL was selected with a sensitivity of 68%. This threshold value seems to be clinically meaningful since 9% of patients without SAE had a C_minPred_ above 2500 ng/mL, vs. 62.5% for patients who experienced SAE.

Drug–drug interactions are a major concern in the management of cancer patients [22] since they often receive multiple drugs to maximize the therapeutic effect, counter the adverse events of chemotherapy, or treat comorbidities. Additionally, they can concomitantly consume herbs, food and dietary supplements that can interact significantly on the PK of anticancer drugs [23]. PDI may result in severe adverse events related to plasma overexposure or decreased efficacy in the case of subtherapeutic concentration. CYP3A4-based drug–drug interactions are often observed in daily clinical practice [22]. Olaparib is a candidate for PDI because it is mainly metabolized through the CYP3A4 pathway. In the present study, the two illustrative cases of PDI with amiodarone highlight the three stages of PDI: CYP3A4 inhibition resulting in plasma drug overexposure and in consequence, the occurrence of an SAE. Overall, these results suggest that a multidisciplinary approach including oncologists, pharmacists and pharmacologists should be conducted to prevent clinically significant drug–drug interactions before the start and during olaparib treatment.

The large inter-individual variability in olaparib plasma exposure observed in our study (CV = 64% and 53% for capsules and tablets, respectively), as well as the PK-toxicity relationship, supports the use of therapeutic drug monitoring strategies in a context of personalized drug management. Our study shows that the application of model-informed therapeutic drug monitoring for olaparib could be considered in routine practice to guide dose adaptations. Indeed, a blood sample for olaparib quantification can be collected at any time after dose intake and a corresponding trough concentration can be estimated using the previously published PK model. However, since this approach is not yet used in all the hospital laboratories, olaparib trough concentration can be obtained by sampling blood at 10 to 14 h after dose intake (considering terminal half-life of 15 h [21]. The proposed threshold of 2500 ng/mL could help to guide dose adaptation in order to prevent the onset of SAE and/or to confirm whether a clinical event is due to olaparib plasma overexposure. Other PK endpoints such as area under the concentration–time curve (AUC) or maximum concentration (C_max_) were not evaluated in this study since we aimed to find a PK target that could be easily obtained in routine care.

Few limitations should be taken into consideration. This threshold value could be under- or overestimated due to the possible biased inclusion of patients for whom plasma drug monitoring was performed because of apparent frailty. In addition, our PK-toxicity analysis was based on C_minPred_ estimated using sparse PK data in real-life patients and a population PK model from the literature. The model was developed based on data from clinical trials and therefore might not represent the unselected patients seen in routine practice. Therefore, the threshold of 2500 ng/mL associated with SAE should be interpreted cautiously and can now be used when the estimation of C_minPred_ is performed with the PK model by Zhou et al. Further validation should confirm this threshold on measured trough concentrations in a larger prospective cohort with standard TDM practice.

Furthermore, the covariates included in the PK model were formulation (capsule or tablet), tablet strength and ECOG-PS (0 vs. 1–2). Although a reduced initial dose of olaparib is currently recommended in patients with renal impairment [24], in two population PK analyses, creatinine clearance (CL_CR_) did not have a statistically significant impact on olaparib elimination (CL/F) [19,25]. Therefore, renal impairment was not included in our estimations. However, other factors such as concomitant intake of CYP3A inhibitors or inducers or inflammation might impact the PK of olaparib [26,27]. The use of a physiologically-based PK (PBPK) model could be more accurate to predict C_minPred_ in such patients. A PBPK model for olaparib has been previously proposed and allows predicting changes in olaparib exposure in various scenarios such as intake of CYP3A modulators and might be more suitable to use in routine practice [17]. In addition, a PBPK model could be used to predict concentrations at the site of action (tumor tissue) for a better description of the PK–efficacy relationship. Future investigations should consider the use of PBPK models for the estimation of olaparib individual exposure.

Finally, recent olaparib approvals in first-line treatment in ovarian and breast cancer [4,28], together with development in combination with other drugs [29,30,31] prompt to anticipate a wide use of this drug in a heterogeneous population. In this context, the development of a pharmacokinetic-guided dosing strategy appears relevant to encompass expected heterogeneous clinical and toxicity profiles. Beyond olaparib, our results suggest that these approaches should be developed also in other PARP inhibitors.

## 4. Materials and Methods

### 4.1. Patients

All consecutive patients who were referred to Cochin University Hospital for olaparib plasma exposure assessment as per routine practice between January 2016 and September 2020 were considered for inclusion in this retrospective cohort. Patients were treated and followed in Paris urban area University Hospitals (Cochin University Hospital, Georges Pompidou European Hospital, Gustave-Roussy Cancer Campus). We included only patients who received olaparib in maintenance for high-grade serous or endometrioid ovarian cancer, primary peritoneal cancer, or fallopian tube cancer, with a *BRCA1/2* mutation, and who had a complete or partial clinical response after platinum-based chemotherapy, regardless of the line of treatment. Exclusion criteria were: minor patients, patients receiving olaparib for other cancers, and patients referred for a second medical opinion and for whom follow-up data were not available. Patient and olaparib therapeutic management was performed according to routine practice.

### 4.2. Data Collection

Patient electronic medical records were retrospectively searched for patients (age, comorbidity), cancer characteristics (International Federation of Gynecology and Obstetrics classification (FIGO), histological type, *BRCA1/2* mutation), and therapeutic management (surgery, previous chemotherapy) at diagnosis. Clinical and biological characteristics were collected at olaparib initiation: body weight, size, functional status (as per Eastern Cooperative Oncology Group Performance Status (ECOG-PS)), hemoglobin, platelets, white blood cells count, kidney function (estimated Glomerular Filtration Rate (eGFR)), and serum albumin concentration. Olaparib dose, galenic formulation (tablet or capsule) and concomitant treatments were also notified. Potential PK drug–drug interactions (PDI) with olaparib were assessed using both the Cancer Drug Interaction website (Radboud UMC and University of Liverpool [32] and DDI Predictor [33]. They were classified in five degrees of interaction, increasing in significance: no clear data, no interaction expected, potential weak interaction, potential interaction, do not co-administer.

Olaparib-induced adverse events were graded using the National Cancer Institute Common Toxicity Criteria (CTCAE), version 5.0.

### 4.3. Pharmacokinetic Analysis

Monitoring of plasma olaparib concentrations was performed as per routine practice at the practitioner’s discretion. Blood samples were collected in heparinized tubes (5 mL) at any time during the dosing interval, then centrifuged at 4000 RPM for 10 min within 2 h after collection. Plasma was collected, then stored at −20 °C until analysis. Quantification of olaparib plasma concentrations was performed using a validated high-performance liquid chromatography (HPLC)-UV method. Briefly, 200 µL of plasma sample (calibration standard, internal quality control (IQC) or patients’ sample) were firstly spiked with 400 µL of erlotinib (250 ng/mL) used as internal standard (IS), then with 400 µL of acetonitrile including 0.1% trifluoroacetic acid (TFA). Samples were vortexed for 10 min, centrifuged (13,000 RPM, 5 min) and the organic layer was evaporated under nitrogen stream at 40 °C. Dry residue was reconstituted with 100 µL of mobile phase (ammonium acetate 20 mM acidified to pH 4.5 with TFA and acetonitrile 0.1% TFA (75:25, *v*/*v*)). The vials were vortexed, then ultracentrifuged (13,000 RPM, 5 min). Finally, 50 µL of supernatant was injected into the chromatographic system. This latter consisted of Dionex Ultimate 300 equipped with a gradient pump with degas option and gradient mixer, a UV-visible detector, an autosampler, and a Chromeleon^®^ chromatography workstation (Dionex Corporation, Sunnyvale, CA, USA). The separation of analytes was performed using a NUCLEOSHELL Bluebird RP 18 (2.7 µm, 150 × 4.6 mm) column (Macherey-Nagel, Düren, Germany) associated with a guard column packed with the same bonded phase. The column temperature was maintained at 40 °C and the auto-sampler at 4 °C. The mobile phase consisted of a mixture of ammonium acetate 20 mM acidified to pH 4.5 with TFA (reagent A) and of acetonitrile 0.1% TFA (reagent B). The mobile phase was delivered using isocratic elution (75% A; 25% B, *v*/*v*) at a flow rate of 1 mL/min. UV detection was performed at 210 nm and 323 for olaparib and erlotinib (IS), respectively. The calibration curve was linear in the range 100–10,000 ng/mL Within-day imprecision and accuracy were for three levels of IQC: 3.0% and −1.1% for 200 ng/mL, 5.6% and −1.8% for 700 ng/mL and 2.7% and 4.7% for 4000 ng/mL, respectively. Between-day imprecision and accuracy were 9.6% and 4.7% for 200 ng/mL, 6.8% and 2.5% for 700 ng/mL and 6.5% and 6.7% for 4000 ng/mL, respectively. The lower limit of quantification (LLOQ) was 100 ng/mL with between-day imprecision and accuracy of 7.4% and −1.2%, respectively.

Since PK sampling was performed at any time during the dosing interval, a previously published population PK model for olaparib was used to predict trough concentrations (i.e., 12 h post-dose, C_minPred_) [19]. Briefly, the model consisted of two compartments for olaparib distribution, sequential zero- and first-order absorption and first-order elimination for both capsules and tablets formulation. To account for the differences in plasma exposure between capsules and tablets formulations, the relative bioavailability (F_rel_) of the capsule and tablets formulation was included in the PK model, with capsule < 100 mg dose as the reference (i.e., F_rel_ = 1). The residual error was coded according to the proportional model and the inter-individual variability was included according to the exponential model. The covariates included in the published PK model and used in the current analysis were: olaparib formulation (capsule or tablet), tablet strength and ECOG-PS (0 vs. 1–2). The PK analysis was performed in NONMEM software version 7.5.0 (ICON Development Solutions, Ellicott City, Maryland). The first-order conditional estimation with interaction (FOCE-I) method was used with MAXEVAL = 0 option with the parameter values fixed to the values estimated in [19]. The PK model parameters are summarized in Table A1.

Predictive performance of the PK model was evaluated by calculating the prediction error (PE) as the difference between the observed and predicted plasma olaparib concentrations according to the following equation: PE_ij_ = (C_pred,ij_ − C_obs,ij_)/C_obs,ij_ × 100 where C_pred,ij_ is the *j*th predicted concentration for individual *i* and C_obs,ij_ is the *j*th observed concentrations for individual *i***.** The accuracy was estimated using the median PE while the precision was measured by the interquartile range (IQR) of the PE. The absolute value of PE was computed and the number of concentrations with absolute PE <20% and <30% was reported. In addition, a prediction-corrected visual predictive check (pcVPC) was performed to evaluate the accordance between the concentrations simulated with mean population parameter values (fixed and random effects) issued from the published model and the observed olaparib concentrations in our study population.

The PK model was used to compute 12-h post-dose plasma olaparib concentrations (trough concentration, C_minPred_) considering each individual concentration separately, which allowed accounting for potential intra-patient variability.

### 4.4. Pharmacodynamic Analysis

#### 4.4.1. Olaparib Toxicity

The primary endpoint of the study was the occurrence of clinically significant adverse events (SAE) considered as olaparib-related (as stated in medical records). No secondary interpretation nor adjudication of causality between olaparib and adverse events was performed. SAEs were defined as: (i) grade III-IV adverse events, or (ii) adverse events resulting in dose reduction or treatment discontinuation.

#### 4.4.2. Statistical Analyses

Patient characteristics were described as number (percentage) for binary/categorical variables and median [interquartile range, IQR] or range (min–max) for continuous variables. Associations between continuous and binary variables were assessed using the non-parametric unpaired Wilcoxon test. Associations between binary/categorical variables were assessed by Fisher or Chi2 test, as appropriate.

The association between patient baseline characteristics and SAE occurring within the first six months of treatment was assessed by logistic regression. The PK-toxicity relationship was assessed based on SAE events for which olaparib plasma concentration (C_minPred_) close to the onset (+/− 6 months) was available. In patients who experienced an SAE, C_minPred_ closest to SAE was considered for statistical analysis. In patients who did not present any SAE, the mean plasma exposure was calculated by averaging all available C_minPred_ for each patient. The association between C_minPred_ and SAE onset was assessed by logistic regression. Performances of C_minPred_ as a predictor of SAE onset were evaluated by area under the ROC curve (AUC) analyses. We aimed to identify a C_minPred_ threshold with high specificity (high predictive positive value for olaparib toxicity), to suggest dose reduction only in patients with high confidence of over-exposure. All tests were two-sided. Variables reaching *p* < 0.1 in univariable logistic regression analyses were considered eligible for multivariable analysis. Statistical significance was defined by *p* < 0.05. Statistical analyses were performed using *R* software (v4).

## 5. Conclusions

Our study shows an association between olaparib plasma exposure and toxicity in patients treated for *BRCA1/2* mutated ovarian cancer. Although further validations are warranted to confirm or refine the threshold of C_minPred_ identified as indicative of SAE, our results suggest that personalized olaparib drug monitoring is feasible in this setting, and should be considered to guide therapeutic decisions of dose adjustment.

## Figures and Tables

**Table 1:** Patient and cancer characteristics.

Variable	Value
**Patients and cancer characteristics at diagnostic**	
Age, median [Q1–Q3] (years), (27 ^‡^)	59 [53–66]
Histological subtypes, N (%), (27 ^‡^)	
High grade ovarian serous carcinoma	25 (93%)
Others	2 (7%)
FIGO stage at diagnosis, N (%), (27 ^‡^)	
I	2 (8%)
III	22 (81%)
IVB	3 (11%)
*BRCA* mutations ^§^, N (%), (27 ^‡^)	
*BRCA1*	19 (70%)
*BRCA2*	8 (30%)
Initial therapeutic management, N (%), (27 ^‡^)	
Induction/neoadjuvant platinum-based chemotherapy	14 (52%)
Cytoreductive surgery	25 (93%)
Complete resection achieved	23 (85%)
Adjuvant platinum-based chemotherapy	25 (93%)
Patient characteristics at olaparib initiation	
Age, median [Q1–Q3] (years), (27 ^‡^)	63 [57–72]
ECOG-PS, N (%), (27 ^‡^)	
0	5 (19%)
1	16 (59%)
2	6 (22%)
Body mass index (kg/m^2^), median [Q1–Q3], (25 ^‡^)	23 [20–26]
Hemoglobin (g/dL), median [Q1–Q3], (26 ^‡^)	11.8 [11.1–12.4]
White blood cells count (G/L), median [Q1–Q3], (25 ^‡^)	5.1 [3.6–6.2]
Platelet count (G/L), median [Q1–Q3], (26 ^‡^)	233 [187–288]
Serum albumin (g/L), median [Q1–Q3], (20 ^‡^)	42 [39–44]
Estimated creatinine clearance (Cockcroft–Gault formula), median [Q1–Q3] (mL/min) (26 ^‡^)	78 [54–97]
**Cancer characteristics at olaparib initiation**	
Olaparib introduction setting, N (%), (27 ^‡^)	
Maintenance after adjuvant chemotherapy	7 (26%)
First relapse	12 (44%)
Beyond first relapse	8 (30%)
Number of metastatic sites, N (%), (27 ^‡^)	
Complete remission at olaparib initiation	10 (37%)
1	11 (40%)
2	5 (19%)
3	1 (4%)
Metastatic sites, N (%), (27 ^‡^)	
Peritoneal metastases	15 (55%)
Node metastases	4 (15%)
Visceral abdominal metastases	3 (11%)
Extra abdominal metastases	2 (7%)
Olaparib formulation and dosing, N (%), (27 ^‡^)	
Capsule 400 mg bid	16 (59%)
Capsule 200 mg bid	1 (4%)
Tablet 300 mg bid	10 (37%)
**Total**	**27 (100%)**

^‡^ Number of patients with available data. FIGO: International Federation of Gynecology and Obstetrics (FIGO) classification (2014). ^§^ the somatic or germline status was not determined for all patients. ECOG-PS: Eastern Cooperative oncology Group Performance status.

**Table 2:** Association between baseline patient characteristics and risk of olaparib toxicity.

Categories	SAE within 6 Months * Odds Ratio † [95% CI]	*p*-Value †
Age at olaparib initiation (years), (27 ^‡^), for each additional year	1.01 [0.99; 1.03]	0.13
ECOG-PS > 1, (27 ^‡^)	2.66 [0.42; 22.5]	0.31
Body mass index (kg/m^2^), (25 ^‡^), for each additional unit	1.03 [0.99; 1.07]	0.10
Serum albumin (g/L), (20 ^‡^), for each additional unit	0.95 [0.91; 1.00]	0.10
Renal insufficiency, (27 ^‡^), Cockcroft–Gault estimated clearance < 60 mL/min	0.77 [0.15; 3.85]	0.75
Hemoglobin (g/dL), (26 ^‡^), for each additional unit	0.93 [0.72; 1.18]	0.57
Olaparib formulation (27 ^‡^), capsule (reference) vs. tablet	2.14 [0.44; 11.3]	0.34
Olaparib introduction setting (27 ^‡^), maintenance (reference) vs. first relapse and beyond	0.26 [0.03; 1.57]	0.16

* SAE: significant olaparib-related adverse event defined by (i) grade III–IV adverse events, or (ii) adverse events resulting in dose reduction or treatment discontinuation. † *p*-value and odds-ratio computed using univariable logistic regression analysis. ^‡^ Number of patients with available data included in the logistic regression analysis. ECOG-PS: Eastern Cooperative oncology Group Performance status.

**Figure A2:**
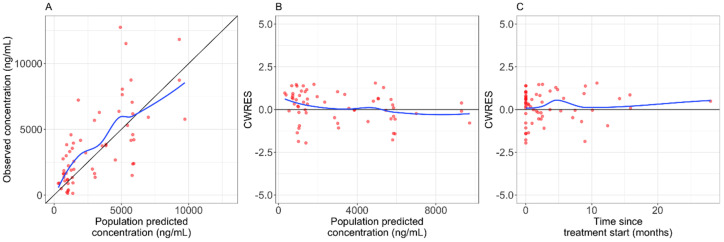
Goodness-of-fit plots. (**A**) Observed vs. population predicted concentrations; (**B**) Conditional weighted residuals vs. population predicted concentrations; (**C**) Conditional weighted residuals vs. time since treatment start. The blue line is the local regression.

## Data Availability

Data is contained within the article.

## Appendix A

**Table A1:** Parameters used in the PK analysis obtained from Zhou et al. [19].

Parameter	Description	Value Used in the Predictions (Mean Estimate from Zhou et al.)
CL (L h^−1^)	Steady-state clearance	3.60
θ_ECOG-PS_ on CL	Effect of ECOG-PS on clearance for ECOG-PS ≥ 1 ^a^	−0.240
V_c_ (L)	Volume of distribution of the central compartment	2.57
V_p_ (L)	Volume of distribution of the peripheral compartment	19.7
Q (L h^−1^)	Intercompartmental clearance	1.11
F_rel_ capsule ^b^	Relative bioavailability for capsule > 100 mg dose	0.282
F_rel_ tablet ^c^	Relative bioavailability for tablet formulation	0.627
k_a_ (h^−1^) capsule	First-order absorption rate constant for capsules	0.247
k_a_ (h^−1^) tablet strength 100 mg	First-order absorption rate constant for tablets strength 100 mg	0.374
k_a_ (h^−1^) tablet strength 150 mg	First-order absorption rate constant for tablets strength 150 mg	0.267
D_1_ (h) capsule	Duration of zero-order absorption for capsules	0.901
D_1_ (h) tablet	Duration of zero-order absorption for tablets	0.467
ω CL (CV%)	Inter-individual variability in CL	0.340 (58.3)
ω V_c_ (CV%)	Inter-individual variability in V_c_	0.448 (66.9)
ω V_p_ (CV%)	Inter-individual variability in V_p_	1.44 (120)
ω Q (CV%)	Inter-individual variability in Q	0.560 (77.2)
ω D_1_ (CV%)	Inter-individual variability in D_1_	0.534 (73.1)
ω k_a_ (CV%)	Inter-individual variability in k_a_	0.063 (25.1)
Proportional residual error	Residual unexplained variability	0.354

CV, coefficient of variation; ECOG-PS, Eastern Cooperative Oncology Group Performance Status. ^a^ reference: ECOG-PS = 0, coded as TVCL = θ_CL_ × (1 + θ_ECOG-PS_). Of note, in the published model, ECOG-PS was coded as 0 (reference), 1 and 2. In our analysis, ECOG-PS = 1 and 2 were gathered into one category because of the limited number of patients and the clinical variability in terms of functional status assessment depending on clinicians. ^b^ relative to capsule < 100 mg dose (i.e., F_rel_ = 1), coded as TVF1 = 1−θ_Frel,capsule_ × LOG10(DOSE). ^c^ relative to capsule < 100 mg dose (i.e., F_rel_ = 1).
